# Enhanced Visible-Light Photocatalytic Activity of TiO_2_ via LaFeO_3_ Perovskite Modification

**DOI:** 10.3390/nano16140880

**Published:** 2026-07-17

**Authors:** Perizat Zhanbirbayeva, Ainur Kayumova, Nazym Yermek, Askhat Baltabekov, Olzat Toktarbaiuly, Nurxat Nuraje, Timur Serikov

**Affiliations:** 1 Faculty of Physics and Technology, Karaganda National Research University Named After Academician Ye. A. Buketov, Karaganda 100026, Kazakhstan; 2 National Laboratory Astana, Nazarbayev University, Astana 010000, Kazakhstan; 3 Institute of New Materials and Energy Technologies, Nazarbayev University, Astana 010000, Kazakhstan; 4 Department of Chemical & Materials Engineering, School of Engineering & Digital Science, Nazarbayev University, Astana 010000, Kazakhstan

**Keywords:** TiO_2_, LaFeO_3_, nanocomposite, perovskite, photocatalysis, photocurrent

## Abstract

In this work, TiO_2_/LaFeO_3_ nanocomposites with varying lanthanum ferrite content were synthesized and comprehensively characterized to enhance the efficiency of photocatalytic processes under solar irradiation. The structural, morphological, and textural properties of the samples were investigated by SEM, TEM, HRTEM, XPS, Raman spectroscopy, and BET analysis. The incorporation of LaFeO_3_ was found to extend the light absorption range of TiO_2_ into the visible region and to modify the surface textural characteristics while preserving the mesoporous structure of the composites. Photoelectrochemical measurements and electrochemical impedance spectroscopy revealed that the formation of a p–n heterojunction between n-TiO_2_ and p-LaFeO_3_ promotes more efficient separation of photogenerated charge carriers and significantly reduces the charge-transfer resistance. The best performance was achieved for the TLFO_2.0 sample, which exhibited the highest photocurrent density (51.85 μA/cm^2^) and the highest photocatalytic activity toward the degradation of MB, RhB, and CR. After 240 min of irradiation, the degradation efficiencies reached 57.3% for MB, 90% for RhB, and 33.8% for CR. These results demonstrate that optimizing the LaFeO_3_ content is an effective strategy for enhancing the photoelectrochemical and photocatalytic performance of TiO_2_/LaFeO_3_ nanocomposites, highlighting their promise for applications in wastewater treatment and solar energy conversion systems.

## 1. Introduction

The growth of the global population and the expansion of industrial production lead to excessive consumption of electrical energy, which is accompanied by the emission of hazardous pollutants, exerting a negative impact on the environment [1]. Consequently, developing alternative energy sources that ensure sustainable development with minimal environmental footprint has become a critical global priority. Among these, solar energy represents one of the most promising solutions due to its abundance, renewability, and environmentally benign nature [2]. However, despite its widespread application in photovoltaics, solar collectors, and thermal power plants, its contribution remains negligible compared to global energy consumption [3,4,5]. To address this, photocatalysis has emerged as an efficient, sustainable approach for simultaneous environmental remediation and solar energy conversion. This technology enables the complete mineralization of organic pollutants in aqueous systems, while demonstrating significant potential for H_2_ production and CO_2_ reduction [6].

Contemporary research in photocatalysis focuses heavily on developing highly efficient materials capable of harvesting solar light and driving photoelectrochemical reactions [7,8,9]. The efficiency of these processes is fundamentally governed by the intrinsic properties of the photocatalyst, such as its band gap energy, the recombination rate of photogenerated charge carriers, and the specific surface area. Among the most viable and widely investigated photocatalytic materials are ZnO [10], SnO_2_ [11], TiO_2_ [12], Cu_2_O [13], WO_3_ [14], SrTiO_3_ [15], BiVO_4_ [16], CdS [17], Fe_2_O_3_ [18], NaTaO_3_ [19], g-C_3_N_4_ [20], MoS_2_, graphene, and its derivatives [21]. Among these materials, TiO_2_ remains one of the most extensively studied and widely used photocatalysts owing to its excellent chemical and photochemical stability, low cost, environmental friendliness, and the versatility of its crystal structure and surface chemistry, which can be tailored to enhance photocatalytic performance [22,23]. However, its practical application is significantly limited by its wide band gap, which restricts visible-light absorption, as well as by the rapid recombination of photogenerated electron–hole pairs. These limitations have stimulated extensive research into various strategies for improving the photocatalytic performance of TiO_2_, including metal and non-metal doping, surface engineering, and the fabrication of composite and heterostructured materials [24,25,26].

Among the various modification strategies, the construction of TiO_2_-based composites with perovskite oxides ABO_3_ has attracted considerable attention because of their ability to extend light absorption into the visible region and promote more efficient charge separation and transfer [27,28]. Perovskites with the general formula ABO_3_ permit substitution at both the A-site (typically rare-earth or alkaline-earth elements) and the B-site (transition metals). This enables precise tuning of their electronic structures, oxidation states, and oxygen vacancy concentrations—factors that crucially dictate the material’s photocatalytic performance. TiO_2_ has been successfully coupled with various perovskite materials, including halide perovskites (CsPbI_3_, CsPbBr_3_, MAPbI_3_, and FAPbI_3_) [29,30,31,32], oxide perovskites (LaFeO_3_, BiFeO_3_, and YFeO_3_) [33], manganites (LaMnO_3_, PrMnO_3_, and NdMnO_3_) [34,35], and cobaltites (LaCoO_3_ and SmCoO_3_) [36,37], resulting in enhanced visible-light harvesting and improved photocatalytic performance. Among these materials, LaFeO_3_ has attracted particular attention because of its relatively narrow band gap (~2.0 eV), excellent thermal and photochemical stability, and favorable electronic structure for visible-light photocatalysis [38]. Notably, LaFeO_3_-based photocatalysts have demonstrated excellent photostability over 15 h of continuous irradiation across six consecutive cycles [39,40]. As a p-type semiconductor, LaFeO_3_ forms a heterojunction characterized by a built-in electric field when brought into contact with n-type TiO_2_. Under light excitation, photogenerated electron–hole pairs are produced in both domains, driving electrons to migrate to TiO_2_ while holes remain localized in LaFeO_3_. This behavior facilitates efficient charge separation, suppresses recombination, prolongs carrier lifetimes, and enhances photocatalytic performance by expanding light harvesting into the visible spectrum [41,42].

A comprehensive analysis of published data reveals that the overall photocatalytic efficiency is strongly governed by the synthesis methodology and the specific concentration of LaFeO_3_ within the TiO_2_ matrix. For instance, incorporating sol–gel-derived LaFeO_3_ nanoparticles into the TiO_2_ framework was shown to play a crucial role in suppressing electron–hole pair recombination, thereby improving methylene blue degradation during photosonocatalytic processes [43]. In another study, LaFeO_3_ loadings ranging from 2.5 to 25 wt.% in TiO_2_ nanoparticles were evaluated for the photocatalytic oxidation of the pesticide myclobutanil; under simulated solar light, complete mineralization of the contaminant was achieved within 240 min for the 5 wt.% and 12.5 wt.% samples [44]. Furthermore, the influence of sol–gel synthesized spherical LaFeO_3_ nanoparticles on the ferromagnetic and photosonocatalytic activity of TiO_2_ was investigated, confirming that LaFeO_3_ integration substantially accelerates the light-induced degradation rate of methylene blue [45]. The photocatalytic performance of TiO_2_/LaFeO_3_ nanocomposites prepared via a facile liquid chemical route has also been explored; here, the photocatalytic degradation of gaseous acetaldehyde and aqueous phenol was found to depend directly on the generation yield of OH radicals, which was controlled by adjusting the molar mass and the TiO_2_-to LaFeO_3_ ratio [46]. Additionally, TiO_2_/LaFeO_3_ nanocomposites fabricated via a simple solid-state reaction were evaluated for the hydrogen evolution reaction (HER) in both acidic and alkaline media. The resulting architecture exhibited a hydrogen evolution efficiency 5.3 times higher than that of commercial P25 TiO_2_, while maintaining exceptional stability under simulated solar light illumination [47].

Dhinesh et al. investigated the photocatalytic activity of LaFeO_3_/TiO_2_ nanocomposites prepared via a hydrothermal method, demonstrating that the integration of LaFeO_3_ extends light absorption into the visible region and suppresses charge carrier recombination [48]. Consequently, experimental studies confirm that the photocatalytic degradation efficiency of TiO_2_/LaFeO_3_ nanocomposites significantly outperforms that of the individual components, which is attributed to improved charge separation and enhanced visible-light harvesting. However, the efficiency of this process is strongly governed by the fabrication methodology and the specific composition ratio of the composite components. At an optimal LaFeO_3_ content, a balance is struck between extended light absorption and the preservation of efficient charge transport pathways, whereas an excessive amount of the perovskite phase may lead to surface shielding of TiO_2_ and an increased probability of charge recombination.

Despite existing reports devoted to TiO_2_/LaFeO_3_ nanocomposites, the precise influence of the optimal LaFeO_3_ content on the charge transfer mechanism and photocatalytic activity of hydrothermally synthesized composites remains insufficiently understood, which defines the relevance of the present study.

The objective of this work is to determine the optimal amount of hydrothermally synthesized LaFeO_3_ perovskite nanoparticles within the TiO_2_ to achieve maximized photocatalytic performance.

In this study, LaFeO_3_ nanoparticles prepared via a hydrothermal route were incorporated into TiO_2_ at various molar ratios. The successful formation of the nanocomposite materials was comprehensively confirmed, and their photocatalytic activity was systematically evaluated via photocurrent response and the photodegradation of model organic dyes.

## 2. Materials and Methods

All chemical reagents and materials used in this work were of analytical grade and used as received without further purification. Most reagents were purchased from Sigma-Aldrich (St. Louis, MO, USA), including La(NO_3_)_3_ 6H_2_O (99.999%), Fe(NO_3_)_3_9H_2_O (≥99.95%), C_6_H_8_O_7_H_2_O, ammonium hydroxide, TiO_2_, FTO glass substrates (fluorine-doped tin oxide, 7 Ω/cm^2^), rhodamine B (≥95% purity, powder), methylene blue (≥82% purity, powder), and Congo red (≥85% purity, powder).

### 2.1. Synthesis of LaFeO_3_ Perovskite

A total of 5 mmol of La(NO_3_)_3_ 6H_2_O and 5 mmol of Fe(NO_3_)_3_ 9H_2_O were dissolved in 10 mL of deionized water under continuous stirring. Subsequently, 10 mmol of citric acid was added to the solution as a chelating agent, and the resulting mixture was stirred continuously for 2 h at room temperature to ensure complete complexation. The pH of the solution was then adjusted to 9 using ammonium hydroxide, followed by an additional 1 h of stirring to achieve a homogeneous sol. The resulting solution was transferred into a 50 mL Teflon-lined stainless-steel autoclave and subjected to hydrothermal treatment at 180 °C for 12 h. The obtained precipitate was collected by centrifugation, washed several times with deionized water and ethanol to remove residual ions and organic byproducts, and dried at 80 °C for 5 h. Finally, the dried powder was calcined in air at 800 °C for 6 h to obtain crystalline LaFeO_3_.

### 2.2. Synthesis of TiO_2_/LaFeO_3_ Nanocomposites

TiO_2_/LaFeO_3_ (TLFO) nanocomposites were prepared via a co-dispersion method using ethanol as the solvent. Initially, 1 mmol of TiO_2_ powder was dispersed in 4 mL of ethanol under continuous stirring. LaFeO_3_ was then added to the suspension in varying molar amounts (0.5, 1.0, 1.5, 2.0, and 2.5 mmol), while the TiO_2_ content was kept fixed at 1 mmol; the resulting samples were denoted as TLFO_x (x = 0.5, 1.0, 1.5, 2.0, and 2.5). The mixtures were magnetically stirred for 2 h to ensure homogeneous distribution of the components, followed by ultrasonication for 1 h to break up agglomerates and improve particle dispersion. The resulting uniform suspensions were deposited onto fluorine-doped tin oxide (FTO) glass substrates by spin-coating at a rotation speed of 3000 rpm. After deposition, the films were thermally treated in a muffle furnace at 500 °C for 2 h.

The surface morphology and microstructural features of the samples were examined by scanning electron microscopy (SEM) and transmission electron microscopy (TEM). SEM measurements were carried out on a Mira 3 LMU microscope (Tescan, Brno, Czech Republic), while TEM and high-resolution TEM (HRTEM) analyses were performed on a JEM-1400 Plus microscope (JEOL, Tokyo, Japan) operated at an accelerating voltage of 120 kV. X-ray photoelectron spectroscopy (XPS) was conducted using an Axis Ultra DLD spectrometer (Kratos Analytical, Stretford, UK) equipped with a monochromatic Al*K* radiation source (hν = 1486.7 eV, 150 W). The binding energy scale was calibrated against the core-level energies of Au 4*f*_5/2_ (83.96 eV), Ag 3d_5/2_ (368.21 eV), and Cu 2p_3/2_ (932.62 eV) obtained from pure metallic gold, silver, and copper standards. The optical properties of the nanocomposite films were characterized by UV–Vis absorption spectroscopy using a UV-2600i Plus spectrophotometer (Shimadzu, Kyoto, Japan) in the wavelength range of 300–800 nm. Raman spectra were recorded on a Confotec MR520 spectrometer (SOL Instruments, Minsk, Belarus) using an excitation wavelength of λexc = 532 nm. X-ray diffraction patterns were obtained on a multifunctional X-ray diffractometer MiniFlex-600 (Rigaku, Tokyo, Japan). The specific surface area, pore volume, and pore size distribution were determined by the Brunauer–Emmett–Teller (BET) and Barrett–Joyner–Halenda (BJH) methods using an Altamira AMI-200 analyzer (Altamira Instruments, Cumming, GA, USA).

The photoelectrochemical properties of the samples were investigated by electrochemical impedance spectroscopy (EIS) in a two-electrode configuration. The working electrode consisted of a TLFO film deposited on an FTO substrate, while the counter electrode was a platinum layer deposited on FTO (Solaronix, Aubonne, Switzerland). The two electrodes were assembled using a 25 μm-thick polymer spacer (Meltonix, Solaronix, Switzerland), with an iodide/triiodide electrolyte (Iodolyte Z-150, Solaronix, Switzerland) employed as the redox mediator. Impedance spectra were recorded over a frequency range of 1 MHz to 100 mHz at an AC amplitude of 20 mV. The photocatalytic activity of the samples was evaluated through photocurrent response measurements and the photodegradation of organic dyes, specifically methylene blue (MB), Congo red (CR), and rhodamine B (RhB). These dyes serve as model pollutants with distinct surface charges in solution, which significantly influence their adsorption behavior on the photocatalyst surface. In aqueous media, MB behaves as a cationic dye, CR exhibits anionic character, whereas RhB can transition between cationic and anionic states depending on solution pH. This diverse set of dyes helps rule out potential artifacts in the measured photocatalytic efficiency arising solely from electrostatic adsorption effects at the material interface. Furthermore, as these dyes are widely used as benchmarks for evaluating similar photocatalytic systems, their use facilitates direct comparison of the present results with previously published literature.

The photodegradation efficiency was monitored by tracking changes in the optical density (absorbance) of the solutions at the wavelengths corresponding to the respective absorption maxima of the dyes: 662 nm for MB, 498 nm for CR, and 522 nm for RhB. Prior to light irradiation, all samples were immersed in the respective dye solutions for 12 h in the dark to establish adsorption–desorption equilibrium, thereby eliminating the contribution of physical adsorption to the overall degradation rate. The 12 h duration required to reach adsorption–desorption equilibrium was determined experimentally, following the procedure described in our previous study [49]. After this period, the photocatalyst surface under these experimental conditions can be considered saturated with adsorbed dye molecules, and no significant further increase in adsorption is expected. Therefore, the subsequent decrease in dye concentration after turning on the light source is primarily due to the photocatalytic decomposition process, rather than ongoing adsorption.

Photocurrent measurements were performed in a standard three-electrode configuration with an active electrode area of 1 cm^2^, using a CS350 potentiostat equipped with an EIS module (Corrtest Instruments, Wuhan, China); a platinum foil and an Ag/AgCl electrode served as the counter and reference electrodes, respectively. Measurements were carried out in 0.1 M NaOH electrolyte within a photoelectrochemical cell equipped with a quartz window. Photocatalytic experiments were conducted under simulated solar irradiation using a G2009A1 solar simulator (Ossila, Sheffield, UK) under AM 1.5G conditions at a light intensity of 1000 W/m^2^.

## 3. Results and Discussion

**Figure 1:**
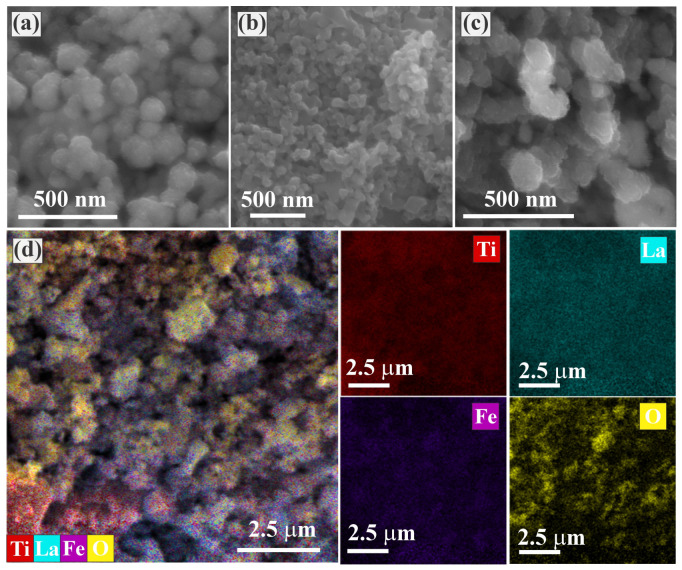
Surface morphology and elemental mapping analysis (**d**) of LaFeO_3_ (**a**), TiO_2_ (**b**), and TLFO_2.0 (**c**) nanocomposites obtained by SEM-EDS.

Figure 1 presents the morphology and elemental mapping analysis of the investigated samples, as revealed by SEM and EDS. The SEM image of TiO_2_ (Figure 1a) reveals that the particles form secondary agglomerates, which can be attributed to van der Waals interactions and the high surface energy typical of nanodispersed systems. At higher magnification, the TiO_2_ nanoparticles exhibit well-defined boundaries and a uniform, quasi-spherical morphology with an average diameter of 20–25 nm (Figure 1a).

LaFeO_3_ nanoparticles exhibit a polydisperse size distribution with an approximately spherical shape and particle sizes ranging from 60 to 100 nm (Figure 1b). The corresponding images indicate that these particles are less agglomerated and possess relatively smooth surfaces compared to TiO_2_. Upon formation of the TLFO nanocomposite (Figure 1c) and subsequent thermal treatment at 500 °C, a densely packed structure with minimal intergranular spacing is observed. The micrographs reveal that, in certain regions, the particles form agglomerates composed of smaller TiO_2_ nanoparticles interspersed with larger LaFeO_3_ particles. Partial coalescence between adjacent particles is also observed, suggesting the initial stages of sintering during thermal treatment and the formation of a well-integrated composite structure. To verify the chemical purity and assess the spatial distribution homogeneity of the components within the TLFO nanocomposite, energy-dispersive X-ray spectroscopy (EDS) elemental mapping was performed (Figure 1d). The overlaid elemental map, along with the individual mappings, demonstrates a highly uniform spatial distribution of all key constituent elements across the analyzed region. These findings confirm the successful formation of a homogeneous heterostructure and underscore the high chemical uniformity of the synthesized nanocomposite.

**Figure 2:**
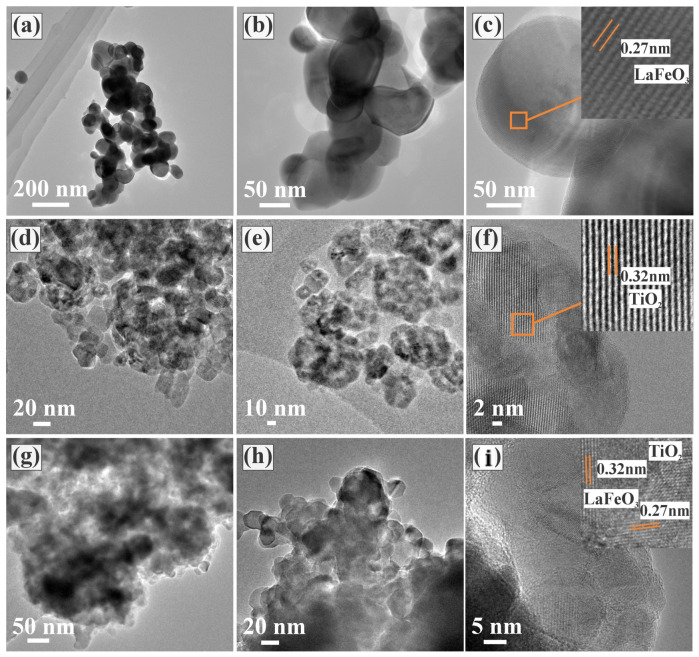
TEM and HRTEM characterization of LaFeO_3_ (**a**–**c**), TiO_2_ (**d**–**f**), and TLFO_2.0 (**g**–**i**) nanocomposites.

TEM images of LaFeO_3_ (Figure 2a,b) confirm that the sample consists of agglomerated, quasi-spherical nanoparticles with strong interparticle contact. HRTEM analysis (Figure 2c) reveals well-resolved lattice fringes with an interplanar spacing of 0.27 nm, corresponding to the crystallographic planes of LaFeO_3_, in good agreement with previously reported values [50]. TEM images of TiO_2_ (Figure 2d,e) confirm the formation of spherical nanoparticles, while the corresponding HRTEM image (Figure 2f) exhibits lattice fringes with an interplanar spacing of 0.32 nm, characteristic of the rutile crystalline phase of TiO_2_.

For the TLFO nanocomposite, TEM images (Figure 2g,h) demonstrate close integration of the two phases, with interconnected TiO_2_ and LaFeO_3_ nanoparticles forming a unified heterogeneous structure. The most compelling evidence is provided by the HRTEM image of the composite (Figure 2i), where two distinct lattice spacings are simultaneously observed: 0.27 nm, corresponding to LaFeO_3_, and 0.32 nm, corresponding to TiO_2_. The coexistence of both crystal lattices within the same region confirms the successful formation of the TiO_2_/LaFeO_3_ heterostructure and the presence of intimate interfacial contact between the components.

**Figure 3:**
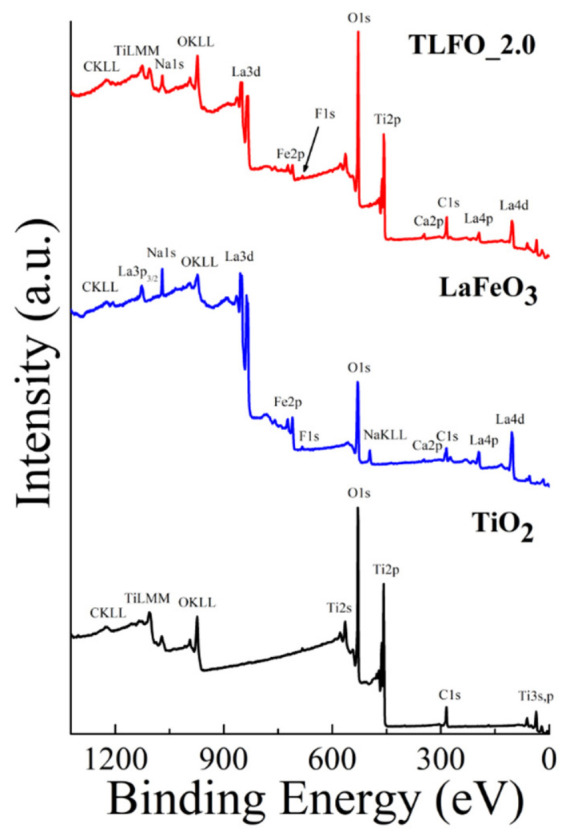
The survey XPS spectrum of TiO_2_, LaFeO_3_ and TLFO_2.0 nanocomposite.

The survey XPS spectrum of the TLFO nanocomposite (Figure 3) reveals characteristic peaks corresponding to Fe, La, Ti, O, C, Ca, Na, and F. The corresponding elemental concentrations, calculated from the survey spectrum, are summarized in Table 1.

**Figure 4:**
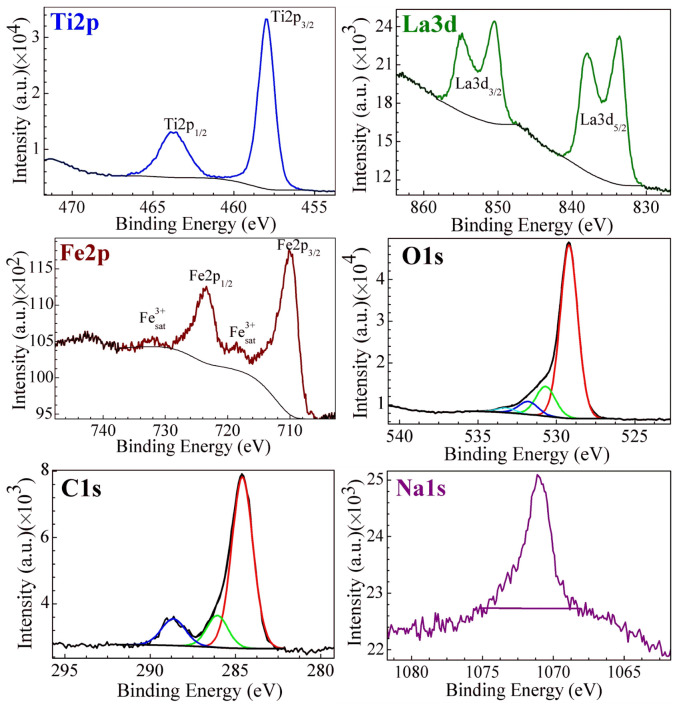
High-resolution XPS spectra.

The Ti2p XPS spectrum (Figure 4) exhibits a characteristic doublet structure, with the Ti2p_3/2_ component located at a binding energy of 458.2 eV, consistent with reported values for TiO_2_. The La3d spectrum displays a well-defined multiplet structure with pronounced shake-up satellites and a La3d_5/2_ binding energy of 833.8 eV, typical of trivalent lanthanum compounds. The Fe2p spectrum features characteristic satellite peaks shifted by approximately 8.5 eV from the main doublet components, indicative of trivalent iron in an oxide environment. In the O1s spectrum, the dominant contribution appears at a binding energy of 529.4 eV, corresponding to lattice oxygen in metal oxides, while additional components arise from surface-adsorbed carbonate, hydroxide, and other oxygen-containing species.

**Figure 5:**
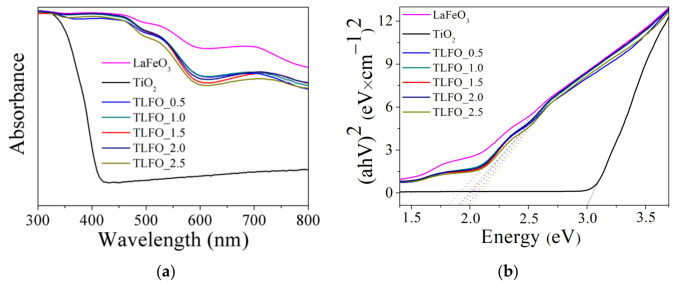
Optical absorption spectra (**a**) and Tauc plots (**b**) of TiO_2_, LaFeO_3_, and TLFO_x nanocomposites with different LaFeO_3_ concentrations.

The absorption spectra of TiO_2_, LaFeO_3_, and the TLFO_x nanocomposites are presented in Figure 5a. Pure TiO_2_ exhibits a sharp absorption edge in the 380–420 nm region, consistent with its wide band gap and predominant absorption in the ultraviolet range; beyond 420 nm, the absorption intensity decreases significantly, confirming its limited activity in the visible region of the spectrum. In contrast, pure LaFeO_3_ nanoparticles exhibit strong absorption across both the ultraviolet and visible regions (300–750 nm), attributed to their narrow band gap. For the TLFO composites, a substantial enhancement in visible-light absorption is observed relative to pure TiO_2_, indicating the successful formation of a heterostructure between TiO_2_ and LaFeO_3_. This enhanced visible-light absorption reflects a synergistic interaction between the two components, which is expected to improve the photocatalytic performance of the resulting materials.

The optical band gap energy (Eg) of the synthesized materials was determined via Tauc plot analysis by a linear extrapolation of the fundamental absorption edge (Figure 5b). For pristine TiO_2_ and LaFeO_3_, the Eg values were found to be 3.0 and 1.8 eV, respectively. As the LaFeO_3_ nanoparticle content within the TiO_2_ matrix increases, the band gap of the TLFO nanocomposites exhibits a slight variation; specifically, the Eg is 1.9 eV for TLFO_0.5 and reaches 2.0 eV for TLFO_2.5, with the remaining samples displaying intermediate values. The observed narrowing of the band gap in the composites relative to pristine TiO_2_ confirms the successful establishment of interfacial electronic coupling, ensuring an efficient optical response of the heterostructures within the visible spectral range.

**Figure 6:**
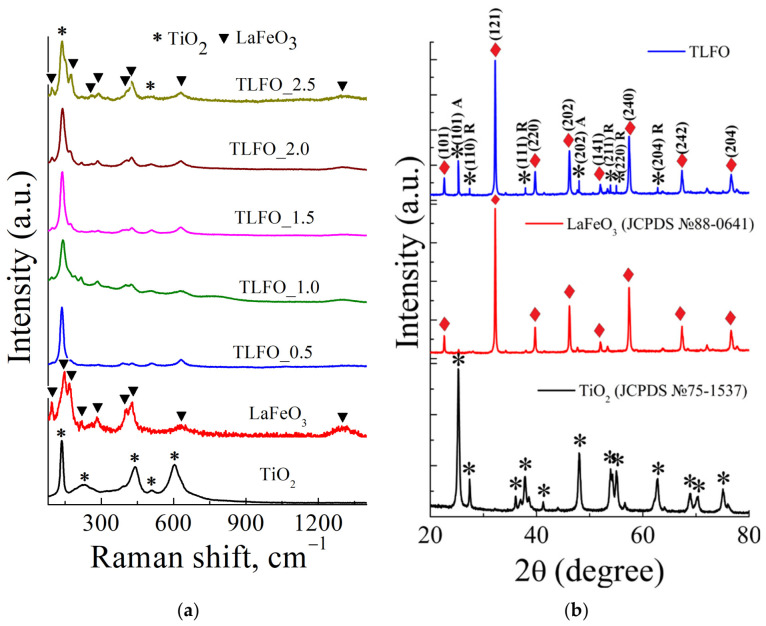
Raman spectra (**a**) and XRD patterns (**b**) of TiO_2_, LaFeO_3_, and TLFO nanocomposite.

The Raman spectra of pristine TiO_2_, LaFeO_3_, and the TLFO_x nanocomposites are presented in Figure 6a. For pure TiO_2_, characteristic lattice phonon modes are observed at 135, 234, 445, 509, and 608 cm^−1^. The low-frequency band at 234 cm^−1^ can be assigned to multiple-phonon scattering or defect-induced vibrations, while the most intense peaks at 445 and 608 cm^−1^ correspond to the fundamental E_g_ and A_1g_ modes of rutile TiO_2_, respectively [51]. The band at 445 cm^−1^ is associated with O–Ti–O bending vibrations, whereas the peak near 608 cm^−1^ is attributed to symmetric Ti–O stretching vibrations. The presence of these well-defined, narrow bands confirms the formation of a highly crystalline rutile TiO_2_ phase.

For LaFeO_3_, Raman peaks are observed at 96, 148, 168, 221, 282, 401, 426, 627, and 1295 cm^−1^. The low-frequency bands at 96, 148, and 168 cm^−1^ are attributed to La^3+^ vibrations and external modes of the orthorhombic perovskite structure of LaFeO_3_. The bands at 221 and 282 cm^−1^ are related to the tilting and deformation of FeO_6_ octahedra, while the signals at 401 and 426 cm^−1^ correspond to Fe–O bending vibrations. The band at 627 cm^−1^ is assigned to symmetric stretching vibrations of FeO_6_ octahedra and serves as a characteristic marker of LaFeO_3_ phase formation [52]. The broad band near 1295 cm^−1^ may be associated with a second-order harmonic overtone or defect-induced vibrations within the structure [53,54].

The Raman spectrum of the TLFO_x composites exhibit characteristic features of both phases, confirming the successful formation of a TiO_2_/LaFeO_3_ heterostructure. The spectrum retains a pronounced band at approximately 445 cm^−1^, corresponding to the E_g_ mode of rutile TiO_2_, along with spectral features characteristic of the perovskite LaFeO_3_ phase. Additionally, an intense broad band is observed around 1295 cm^−1^, indicating a contribution from LaFeO_3_ and a possible increase in structural disorder upon composite formation. Compared to pure TiO_2_, the Raman bands in the composites become broader, and their relative intensities change; these observations can be attributed to interfacial interactions between the two phases, lattice distortion at the TiO_2_/LaFeO_3_ interface, alterations in the length and strength of the Ti–O and Fe–O bonds, as well as an increased concentration of defect states.

The crystalline structure and phase composition of the synthesized samples were investigated by X-ray diffraction (XRD) analysis (Figure 6b). The XRD pattern of pristine TiO_2_ reveals a biphasic structure consisting of a mixture of anatase (A) and rutile (R) phases, in good agreement with JCPDS No. 75-1537. The most intense reflections, located at 2θ 25.3° and 47.9°, correspond to the (101) and (200) crystallographic planes of the tetragonal anatase phase, while the accompanying peaks at 27.4°, 37.8°, 53.8°, 55.0°, and 62.8° match the (110), (111), (211), (220), and (204) planes of the rutile phase.

The diffraction pattern of pristine LaFeO_3_ exhibits a series of sharp, highly intense peaks corresponding to the orthorhombic perovskite structure with space group Pbnm (JCPDS No. 88-0641). The primary reflections are located at 2θ 22.6°, 32.2°, 39.6°, 46.1°, 52.0°, 57.5°, 67.3°, and 76.6°, assigned to the (101), (121), (220), (202), (141), (240), (242), and (204) planes, respectively, with the (121) reflection being dominant.

The XRD pattern of the TLFO_2.0 nanocomposite combines the characteristic peaks of both individual components, confirming the formation of a binary system. Within the composite, both the principal reflections of the orthorhombic LaFeO_3_ perovskite and the diffraction maxima of anatase ((101) at 2θ 25.3° and (200) at 47.9°) and rutile ((110), (111), (211), (220), and (204)) belonging to the TiO_2_ matrix are clearly identified. The absence of extraneous diffraction peaks or noticeable peak shifts indicates the high purity of the synthesized materials, the preservation of the individual crystal lattices of the constituents, and the successful formation of a well-defined TLFO heterostructure. Variations in the LaFeO_3_ content within the TLFO matrix do not induce significant changes in the overall XRD patterns.

**Figure 7:**
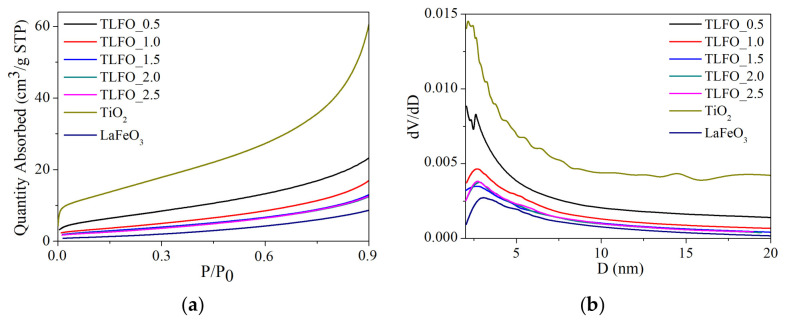
Textural properties of TiO_2_, LaFeO_3_, and TLFO composites obtained by low-temperature nitrogen adsorption: (**a**) adsorption isotherms; (**b**) pore size distribution.

The textural characteristics of the samples were investigated by the BET and BJH methods (Figure 7). According to the IUPAC classification of adsorption isotherms, the isotherms of the investigated samples correspond to Type II, indicating multilayer (multimolecular) adsorption. This isotherm shape is generally characteristic of disperse materials with a corpuscular pore structure formed by the intergrowth of numerous individual particles, in which the pores represent interstitial voids between the intergrown particles. As shown by the adsorption isotherms, the volume of adsorbed nitrogen increases with rising relative pressure (P/P_0_) for all studied samples. The maximum adsorbed gas volume at P/P_0_ = 1.0 is observed for pristine TiO_2_, reaching ~61.9 cm^3^/g, corresponding to a specific surface area (SSA) of 55.12 m^2^/g (Table 2). With increasing LaFeO_3_ content in TiO_2_, both the adsorbed gas volume and the specific surface area decline across the entire pressure range: for TLFO_0.5, the adsorbed gas volume decreases to 0.048 cm^3^/g, and for TLFO_2.5, it drops further to 0.02 cm^3^/g, while the corresponding specific surface area decreases from 26.33 to 11.88 m^2^/g. This behavior is attributed primarily to the size of the synthesized LaFeO_3_ nanoparticles and their concentration within the TiO_2_ matrix, reflecting the inverse relationship between particle size and specific surface area: as particle size increases, the specific surface area inherently decreases. Indeed, the average diameter of the TiO_2_ nanoparticles ranges from 20 to 25 nm, whereas that of the LaFeO_3_ nanoparticles is considerably larger, ranging from 60 to 100 nm. For pristine LaFeO_3_, both the adsorbed gas volume and specific surface area are substantially lower than those of pristine TiO_2_, amounting to 0.014 cm^3^/g and 7.70 m^2^/g, respectively.

Based on the nitrogen adsorption–desorption isotherms measured at 77 K, the pore size distributions of all samples were calculated by the BJH method, and the results are presented in Figure 7b. All studied samples were found to predominantly possess a mesoporous structure, as evidenced by the presence of pores in the 2–10 nm range. The corresponding dV/dD pore size distribution curves reveal that increasing the LaFeO_3_ content in the TiO_2_-based composites leads to a decrease in the number of pores available for gas adsorption.

**Figure 8:**
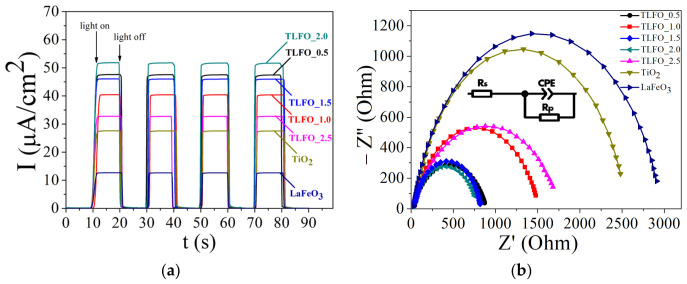
Photocurrent density–time responses (**a**) and Nyquist plots (**b**) of TiO_2_, LaFeO_3_, and TLFO_x nanocomposites with different LaFeO_3_ contents.

Figure 8 presents the photocurrent density–time curves and Nyquist plots for TiO_2_, LaFeO_3_, and the TLFO nanocomposites. As shown in Figure 8a, all investigated samples exhibit a rapid and reproducible photoresponse upon periodic light on/off cycling, indicating stable generation of photoinduced charge carriers and good reversibility of the photoelectrochemical process. Upon illumination, the photocurrent density increases sharply owing to the generation of electron–hole pairs and their subsequent efficient separation under the influence of the built-in electric field and interfacial boundaries; after the light is switched off, the photocurrent rapidly returns to its initial level, confirming the photoinduced nature of the observed signal.

Pure TiO_2_ exhibits a photocurrent density of 27.62 μA/cm^2^, a value typical for TiO_2_ as a photoanode material owing to its high chemical stability and relatively good electron transport properties. However, its photoactivity is limited by its wide band gap, which restricts effective excitation primarily to the ultraviolet region, while bulk and surface charge carrier recombination remain significant factors constraining the photocurrent of pure TiO_2_.

In contrast, pure LaFeO_3_ exhibits a considerably lower photocurrent density of 12.68 μA/cm^2^. Despite its narrower band gap and visible-light absorption capability, the low photoresponse of LaFeO_3_ can be attributed to its lower charge carrier mobility, higher density of defect states, and increased recombination probability. Furthermore, the individual LaFeO_3_ phase lacks an efficient electron-transport framework comparable to that of TiO_2_, which further limits the extraction of photogenerated charges.

Most notably, all TLFO nanocomposites exhibit a higher photocurrent than either individual component, clearly demonstrating the synergistic effect arising from the formation of the TiO_2_/LaFeO_3_ heterostructure. This enhancement is associated not only with the broadened light absorption range but also with more efficient electron–hole separation at the interfacial boundary. The maximum photocurrent is observed for the TLFO_2.0 sample, reaching 51.85 μA/cm^2^—approximately 1.9 times higher than that of pure TiO_2_ and 4 times higher than that of pure LaFeO_3_. This result indicates the existence of an optimal LaFeO_3_ content at which the most favorable combination of factors is achieved: sufficient heterojunction area, extended light absorption, efficient internal charge separation, and preservation of continuous electron transport pathways through TiO_2_.

Figure 8b presents the Nyquist plots for the TiO_2_, LaFeO_3_, and TLFO nanocomposite samples. To systematically investigate the charge transfer and recombination kinetics, the electrochemical impedance spectroscopy (EIS) data were analyzed using the methodology proposed by Adachi et al. [55,56]. The effective electron recombination rate constant ($k_{eff}$) was determined from the peak position of the semicircular arc in the impedance spectrum. According to this model, the angular frequency at the arc maximum is expressed as:

(1) $$\omega_{max}=2\pi f_{max}=k_{eff}$$

where $f_{max}$ is the characteristic frequency corresponding to the apex of the semicircle on the Nyquist plot, and $\omega_{max}$ represents the corresponding angular frequency.

The effective lifetime of the photogenerated electrons was subsequently evaluated as the reciprocal of the recombination rate constant:

(2) $$\tau_{eff}=\frac{1}{k_{eff}}=\frac{1}{2\pi f_{max}}$$

The apex of the impedance arc directly reflects the characteristic electron relaxation frequency, enabling the straightforward quantification of both the recombination rate kinetics and the effective lifetime of the photoinduced charge carriers.

Table 3 summarizes the electrical parameters extracted from the Nyquist plots, including the charge-transfer resistance (R_p_), series resistance (R_s_), effective recombination rate constant (k_eff_), and effective charge carrier lifetime (τ_eff_).

Pure LaFeO_3_ exhibits the highest charge-transfer resistance (R_p_ = 2938.6 Ω), indicating a high recombination rate and low conductivity, while TiO_2_ likewise shows a relatively high resistance (R_p_ = 2477.2 Ω). Among the composites, the lowest R_p_ value is observed for the TLFO_2.0 sample (770.1 Ω), confirming markedly improved charge transfer. This sample also exhibits the lowest effective recombination rate constant (k_eff_ = 1.67 s^−1^) and the longest effective electron lifetime (τ_eff_ = 0.59 s), indicative of suppressed charge carrier recombination. The TLFO_0.5 and TLFO_1.5 samples likewise show reduced resistance relative to the pure components, although their overall characteristics remain inferior to those of TLFO_2.0. A further increase in LaFeO_3_ content (TLFO_2.5) leads to an increase in R_p_ to 1746.1 Ω and a higher k_eff_, suggesting a deterioration of the photoelectrochemical properties caused by excess LaFeO_3_ loading on TiO_2_. The series resistance (R_s_) values for all samples lie within the range of 17.75–59.35 Ω and do not significantly affect the overall trend. Thus, the optimal LaFeO_3_ content in the TiO_2_/LaFeO_3_ composite corresponds to the TLFO_2.0 sample, which exhibits the lowest charge-transfer resistance, the lowest recombination rate, and the longest charge carrier lifetime—characteristics that are directly correlated with its enhanced photocatalytic activity.

**Figure 9:**
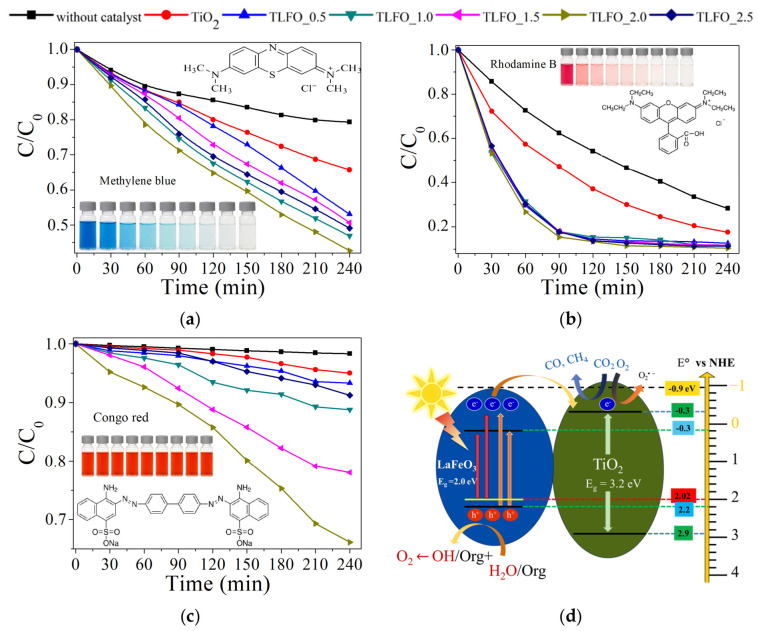
Photocatalytic degradation of organic dyes MB (**a**), RhB (**b**), and CR (**c**) in the presence of TLFO_x samples; (**d**) Schematic representation of the charge separation and transfer mechanism and the photocatalytic degradation processes occurring in the TiO_2_/LaFeO_3_ heterostructure upon light irradiation.

The photocatalytic properties of the nanocomposites were evaluated through the degradation of organic dyes—MB, CR, and RhB. The efficiency of dye photodegradation was assessed by monitoring the change in optical density of the solutions at the wavelengths corresponding to the maximum absorption of each dye, namely 662 nm for MB, 498 nm for CR, and 522 nm for RhB (Figure 9a–c). Prior to the photocatalytic experiments, all samples were immersed in the respective dye solutions for 12 h in the dark to achieve adsorption–desorption equilibrium and to eliminate the contribution of physical adsorption to the overall dye degradation. Under light irradiation in the absence of a photocatalyst, only negligible degradation of all dyes was observed.

As shown in Figure 9a–c, the TLFO_2.0 sample exhibits the highest photocatalytic activity among all synthesized materials. For this composite, the maximum degradation efficiency after 240 min of irradiation reaches 57% for MB, 90% for RhB, and 34% for CR. Samples with lower LaFeO_3_ content exhibit reduced photocatalytic activity, likely due to insufficient formation of interfacial p–n heterojunctions between n-TiO_2_ and p-LaFeO_3_, which are essential for efficient separation of photogenerated charge carriers. A further increase in LaFeO_3_ content (TLFO_2.5) leads to a decline in photocatalytic performance, which can be attributed to a reduction in the number of accessible active sites and hindered charge transfer.

The differences in the degradation rates of MB, RhB, and CR are attributed to the charge state and molecular structure of the dyes. MB and RhB are cationic dyes, whereas CR is anionic; consequently, the cationic MB and RhB molecules are more readily adsorbed onto the photocatalyst surface, facilitating more efficient interaction with photogenerated holes and reactive species (OH^•^ and O_2_^−•^). In contrast, the anionic CR exhibits less favorable adsorption on the composite surface, resulting in a lower degradation rate. The slower photocatalytic degradation of CR may additionally be associated with the high stability of its azo bonds and its more complex molecular structure. The fastest degradation observed for RhB is likely attributable to a combination of effective surface adsorption, photosensitization, and sequential destruction of the organic molecular framework. Overall, these results demonstrate that optimization of the LaFeO_3_ content in TiO_2_-based composites can significantly enhance their photocatalytic activity.

It should be noted that the investigated dyes are widely used in industry and represent typical organic pollutants in wastewater: MB is applied in textile and paper dyeing as well as in analytical chemistry, RhB is used in the textile, paper, and chemical industries, and CR is predominantly employed in the coloration of textiles, paper, and polymeric materials. The high photocatalytic degradation efficiency achieved with the TLFO_x films therefore confirms the potential of the synthesized nanocomposites for wastewater treatment and the removal of persistent organic pollutants.

A schematic illustration of the proposed charge-transfer mechanism and photocatalytic processes occurring in the TiO_2_/LaFeO_3_ heterostructure under light irradiation is presented in Figure 9d.

Upon irradiation, electron–hole pairs are generated in both semiconductors, and the formation of a heterojunction between TiO_2_ and LaFeO_3_ promotes efficient spatial separation of the photogenerated charge carriers. Based on previously reported studies of similar TiO_2_/LaFeO_3_ heterostructures, it can be assumed that electrons may accumulate in the conduction band of TiO_2_, where they are able to reduce molecular oxygen to superoxide radical anions (•O_2_^−^), while holes may remain in the valence band of LaFeO_3_ and react with water molecules or surface hydroxyl groups to generate hydroxyl radicals (•OH) [57].

Accordingly, the reactive oxygen species •O_2_^−^ and •OH may participate in the photocatalytic degradation of organic pollutants, ultimately leading to their mineralization into CO_2_ and H_2_O. The enhanced photocatalytic activity is therefore attributed to more efficient charge separation across the heterojunction, which is believed to suppress charge carrier recombination and prolong the lifetime of photogenerated charge carriers [58].

## 4. Conclusions

In this work, TiO_2_/LaFeO_3_ nanocomposites with varying LaFeO_3_ contents were successfully synthesized, and their structural, optical, textural, photoelectrochemical, and photocatalytic properties were comprehensively investigated. SEM, TEM, and HRTEM analyses confirmed the formation of a TiO_2_/LaFeO_3_ heterostructure with intimate interfacial contact between the components. Raman spectroscopy verified the preservation of the characteristic vibrational modes of rutile TiO_2_ and orthorhombic LaFeO_3_, while XPS analysis confirmed the presence of Ti, La, Fe, and lattice oxygen, further validating successful composite formation.

Optical studies showed that the incorporation of LaFeO_3_ extends the light absorption range of TiO_2_ into the visible region of the spectrum. Textural analysis demonstrated that all samples possess a mesoporous structure; modification of TiO_2_ with lanthanum ferrite leads to a decrease in both nitrogen adsorption and pore volume relative to pure TiO_2_, associated with partial coverage of the TiO_2_ surface by LaFeO_3_ nanoparticles. Nevertheless, the composites retain a well-developed porous structure, which is essential for pollutant adsorption and the transport of reactants to active sites.

The formation of the TiO_2_/LaFeO_3_ heterostructure was found to significantly enhance the separation and transfer of photogenerated charge carriers, as confirmed by the increased photocurrent density and reduced charge-transfer resistance obtained from electrochemical impedance spectroscopy. The highest photoelectrochemical performance was achieved for the TLFO_2.0 sample, which exhibited a photocurrent density of 51.85 μA/cm^2^ and the lowest charge-transfer resistance among all investigated samples. These results point to the existence of an optimal LaFeO_3_ content at which the most favorable combination of extended light absorption, well-developed interfacial contact, and accelerated charge transfer is achieved.

Photocatalytic tests revealed that all TiO_2_/LaFeO_3_ nanocomposites outperform the individual components in the degradation of organic dyes. The highest photocatalytic activity was likewise obtained for the TLFO_2.0 sample, which achieved degradation efficiencies of 57% for MB, 90% for RhB, and 34% for CR after 240 min of irradiation. The higher degradation rates observed for the cationic dyes MB and RhB, compared with the anionic dye CR, are attributed to their more favorable adsorption on the oxide photocatalyst surface, as well as to differences in dye molecular structure.

Overall, optimization of the LaFeO_3_ content proved to be an effective strategy for tailoring the photoelectrochemical and photocatalytic properties of TiO_2_/LaFeO_3_ nanocomposites. The TLFO_2.0 composition represents the most promising formulation, offering an optimal balance of textural properties, extended spectral sensitivity, efficient charge carrier separation, and high photocatalytic activity. These findings confirm the strong potential of TiO_2_/LaFeO_3_ nanocomposites for application in photoelectrochemical systems and in the removal of persistent organic pollutants from wastewater.

## Figures and Tables

**Table 1:** Surface elemental concentrations of the sample (at. %), calculated from survey XPS spectra.

Sample	C	O	Na	La	Fe	F	Ca	Ti
TiO_2_	19.7	57.1	-	-	-	-	-	23.2
LaFeO_3_	21.0	48.6	5.7	14.8	8.6	0.7	0.6	-
TLFO	19.1	53.7	1.6	6.1	2.8	0.6	0.8	15.3

**Table 2:** BET surface area and pore volume of TiO_2_, LaFeO_3_ and TLFO samples.

Sample	S, m^2^/g	V, cm^3^/g
TiO_2_	55.12	0.096
LaFeO_3_	7.70	0.014
TLFO_0.5	26.33	0.048
TLFO_1.0	15.89	0.027
TLFO_1.5	12.55	0.021
TLFO_2.0	12.15	0.019
TLFO_2.5	11.88	0.020

**Table 3:** Electrical characteristics of samples calculated from the impedances of the Nyquist diagram.

Sample	R_p_, Ω	R_s_, Ω	k_eff_, s^−1^	τ_eff_, s
LaFeO_3_	2938.6	25.39	4.25	0.23
TiO_2_	2477.2	59.35	3.36	0.3
TLFO_0.5	867.7	23.68	2.66	0.37
TLFO_1.0	1494.7	20.26	3.36	0.3
TLFO_1.5	791.2	35.58	2.1	0.47
TLFO_2.0	770.1	23.09	1.67	0.59
TLFO_2.5	1746.1	17.75	3.36	0.3

## Data Availability

Data will be available at reasonable requests to the authors.
